# Assessing Genetic Distinctness and Redundancy of Plant Germplasm Conserved Ex Situ Based on Published Genomic SNP Data

**DOI:** 10.3390/plants12071476

**Published:** 2023-03-28

**Authors:** Yong-Bi Fu

**Affiliations:** Plant Gene Resources of Canada, Saskatoon Research and Development Centre, Agriculture and Agri-Food Canada, 107 Science Place, Saskatoon, SK S7N 0X2, Canada; yong-bi.fu@agr.gc.ca

**Keywords:** plant germplasm characterization, genetic distinctness, genetic redundancy, genomic SNP data, average pairwise dissimilarity, germplasm management and conservation, genetic categorization of plant germplasm

## Abstract

Assessing genetic distinctness and redundancy is an important part of plant germplasm characterization. Over the last decade, such assessment has become more feasible and informative, thanks to the advances in genomic analysis. An attempt was made here to search for genebank germplasm with published genomic data and to assess their genetic distinctness and redundancy based on average pairwise dissimilarity (APD). The effort acquired 12 published genomic data sets from CIMMYT, IPK, USDA-ARS, IRRI, and ICRISAT genebanks. The characterized collections consisted of 661 to 55,879 accessions with up to 2.4 million genome-wide SNPs. The assessment generated an APD estimate for each sample. As a higher or lower APD is indicative of more genetic distinctness or redundance for an accession, respectively, these APD estimates helped to identify the most genetically distinct and redundant groups of 100 accessions each and a genetic outlier group with APD estimates larger than five standard deviations in each data set. An APD-based grouping of the conserved germplasm in each data set revealed among-group variances ranging from 1.5 to 53.4% across all data sets. Additional analyses showed that these APD estimations were more sensitive to SNP number, minor allele frequency, and missing data. Generally, 5000 to 10,000 genome-wide SNPs were required for an effective APD analysis. These findings together are encouraging and useful for germplasm management, utilization, and conservation, particularly in the genetic categorization of conserved germplasm.

## 1. Introduction

There are more than seven million plant germplasm accessions of more than 16,500 plant species currently conserved in 1750 genebanks worldwide [1,2], thanks to concerted conservation efforts over the last 60 years. However, it is challenging to manage and conserve these germplasm collections [3,4,5,6]. Large efforts are required to evaluate and characterize these germplasm collections for their conservation and use [7], but insufficient resources are available to genebanks [3,8]. Only two million conserved accessions are estimated to be unique [2]. Consequently, assessing genetic distinctness and/or redundancy has become an important part of germplasm characterization [9,10,11,12,13,14,15,16]. Identification of genetically distinct germplasm can be instructive for the development of core subsets in a germplasm collection (e.g., [17]) and the germplasm selection for safety backup in other genebank facilities, and it can be useful for broadening narrow genetic bases of breeding gene pools (e.g., see [18]). Assessing genetically redundant germplasm can help to identify and validate accession duplication [14,15]. More importantly, genetic categorization of conserved germplasm is needed to enhance current and future germplasm uses.

To facilitate germplasm characterization, we previously developed a genetic marker-based approach using an average pairwise dissimilarity (APD) of an accession against the other assayed accessions to assess genetic distinctness and genetic redundancy in a plant germplasm collection [13]. The APD approach is based on the acquired molecular characterization data, generates the APD estimate of an accession against the remaining assayed accessions, and provides a means to identify genetically distinct or redundant germplasm. A higher APD estimate indicates the accession is more genetically distinct than accessions with lower APD estimates. The approach has been well cited in the scientific literature, but unfortunately, it has not been applied as widely as hoped to assess genetic distinctness and redundancy of conserved germplasm [19].

Genomic characterization of conserved germplasm has become more feasible than before (e.g., see [20,21,22,23]), thanks to the advances in genomic analysis [24]. There are many published genomic SNP data sets for the conserved plant germplasm (e.g., see [21,25]). To take the advantage of the existing genomic data, we attempted to search for conserved germplasm with published genomic data and to assess their genetic distinctness and redundancy based on the APD approach. The effort acquired 12 published data sets with germplasm collections of size ranging from 661 to 55,879 accessions with up to 2.4 million SNPs, representing the genomic characterization of plant germplasm conserved at five major genebanks: CIMMYT, IPK, USDA-ARS, IRRI, and ICRISAT. The assessment generated an APD estimate for each assayed accession. This paper was written to illustrate the application of the APD approach to analyze large genomic SNP data and to publish the acquired APD estimates of the assayed samples for germplasm management. It is our hope that the APD approach is better utilized to facilitate the genetic categorizing of conserved germplasm for more effective germplasm management and utilization.

## 2. Materials and Methods

### 2.1. Acquisition of Published Genomic Data

The SNP genotype and passport data were searched and acquired from publicly available online resources of five major genebanks for conserved germplasm of the following species: *Hordeum vulgare* and *H. spontaneum* ([21]; IPK, Gatersleben, Germany), *Glycine max* and *G. soja* ([25]; USDA-ARS, Fort Collins, USA), *Oryza sativa* ([26]; IRRI, Los Baños, Philippines), *Triticum aestivum* (2n = 6x), *T. durum* (2n = 4x), *T. aethiopicum* (2n = 4x), *Aegilops tauschii* (2n = 2x) and *A. triuncialis* (2n = 2x) ([22]; CIMMYT, Texcoco, Mexico), and *Cicer arietinum* ([23]; ICRISAT, Hyderabad, India) (see Supplementary Table S1). These data sets represented the genomic characterization of plant germplasm conserved at five major seed genebanks: CIMMYT, IPK, USDA-ARS, IRRI, and ICRISAT.

### 2.2. Data Processing

APD estimation per sample would be more informative for samples within a species or species group. Efforts were made to generate 12 specific genomic SNP data sets (Supplementary Table S2) from the acquired genomic SNP data sets (Table S1) and these 12 data sets were named based on their species names for ease of identification. Note that the published rice data set was separated into Oryza sativa Indica and Japonica group data sets, due to their unique genetic features. Overall, these 12 data sets had germplasm collections of size ranging from 661 to 55,879 accessions with up to 2.4 million genome-wide SNPs. APD estimation was performed using a Bioconductor R package SNPRelate [27], which is capable of handling a large genomic SNP data set. Efforts were spent to convert each SNP data set into a VCF or GDS file as required by SNPRelate. As the published SNP data sets were generated for different species of variable ploidy levels using different sequencing technologies by different bioinformatics tools, different data processes were needed to generate the cleaned SNP data sets for the APD analyses. The detailed procedures for data processes to generate each SNP data set were given in the B section of Supplementary Materials.

Briefly, the barley genomic data set is available as separate Hordeum vulgare and Hordeum spontaneum VCF files [21]. The soybean VCF file [25] was split into two separate files (Glycine max and Glycine soja) using BCFtools view (v 1.15.1; [28]), along with the removal of monomorphic loci. The separation was based on the provided passport data and additional passport data collected from various online databases (e.g., USDA-GRIN, PGRC-GRIN-CA). A sample without passport data was assumed to be G. max. This separation was also involved with the conversion of sample labels as described in Supplementary Materials D1 Additional File. Similarly, for rice data, its VCF file [26] was separated into Oryza sativa Indica and Oryza sativa Japonica groups based on the provided passport information using BCFtools view with monomorphic loci removed. The chickpea SNP data set [23] was provided in HapMap format and was converted to VCF format using TASSEL 5 Standalone ([29]; see the processing pipeline described in Supplementary Materials D3 Additional File. Hexaploid, tetraploid, and wild wheat SNP data files [22] were provided in DArT format [30]. These files were subdivided based on the provided passport information, with monomorphic loci removed, and then converted directly to GBS format, retaining only bi-allelic SNP loci, using the dartR R package (See the processing procedures described in Supplementary Materials D2 Additional File). However, the dartR-derived GBS files needed to be transposed to match with the executable SNPRelate GBS file, which was explicitly executed in an R script ADP.r (see its txt file or D4 Additional File of Supplementary Materials) for these data sets.

### 2.3. APD Analysis

Each cleaned data set was first analyzed with respect to allelic frequency, minor allelic frequency, and missing SNP data, allowing for a better understanding of the variability and heterogeneity of these data sets. For each data set, APD and its standard deviation were obtained for each sample using the APD.r script in an R v 4.1.2 environment [31]. The R script was specifically written for this analysis following the method of Fu [13]. Briefly, in a typical marker-based characterization of self-fertile plant germplasm with n samples representing *n* accessions of a collection that are assayed at many SNP loci, a given sample can form *n* − 1 pairs with the remaining assayed samples. For each of such pairs, the genotypic similarity (*S*) can be calculated based on SNP genotypes following the simple matching coefficient of Sokal and Michener [32], and the pairwise dissimilarity is 1 − *S*. The average pairwise dissimilarity (or APD) for the given sample can be obtained by averaging all *n* − 1 pairwise dissimilarity estimates. The higher the APD estimate obtained for the given sample, the more genetically distinct the sample representing the accession is in the collection.

The APD.r script was specifically modified for data input to analyze each of the 12 cleaned data sets (Table S2). Table S2 also provided the number of samples and the maximum number of loci processed, the estimated run times, and the number of computational threads used for each data set. For the computational analysis, a Conda [33] environment with R v 4.1.2 was created on the Agriculture and Agri-Food Canada’s Biocluster high-performance computing platform to run the following R packages: SNPRelate (v. 1.28.0) and dartR (v. 2.0.3) and their dependencies. Our computational capacity did not accommodate an APD analysis of the original published Triticum aestivum and Cicer arietinum data sets, and consequently, we generated new workable data sets with 20,000 and 300,000 SNPs, respectively, that were randomly selected from the original SNP data sets (Table S2).

The acquired APD estimates in each data set were further analyzed for their variations with basic statistics and distribution. Grouping was made on all the samples in a data set based on the distribution (M for mean and SD for standard deviation) of ADP estimates: 1 (M + 3SD), 2 (M + 2SD), 3 (M + SD), 4 (M), 5 (M − SD), and 6 (M − 2SD). Analysis of molecular variance (AMOVA; [34]) was also made on the six APD-based groups using the R package poppr [35] to characterize the APD-group variation. Group-specific Fst estimate was made following the method of Weir and Goudet [36] using SNPRelate.

To facilitate plant germplasm management and utilization, we identified a genetic outlier group of samples with APD estimates larger than five standard deviations and generated genetically distinct and redundant groups with 100 samples in each data set by selecting the samples with the largest or smallest APD estimates, respectively. These two distinct and redundant groups were further characterized for APD variation, along with the published passport data such as origin country and biological status (landrace, cultivar, breeding line, etc.), relative to the whole cleaned data set.

### 2.4. Analysis of APD Estimation with Large Genomic Data

This was the first time that APD estimation was applied to such a large genomic data set, and the APD properties were largely unknown. Efforts were made to assess the impacts of sample size, SNP number, minor allelic frequency, and missing data on APD estimation. Specifically, an APD correlation analysis was performed using a custom R script between the original APD estimates and new APD estimates of the same samples, in which the former was obtained from the whole original data set, and the latter was generated under a scenario with respect to each of the four mentioned variables. A higher correlation means that the APD estimates in the analyzed scenario would provide an APD-based sample ranking more consistent with those from the APD estimates of the original genomic data set.

For sample size, we focused on two large data sets, Hordeum vulgare and Glycine max, and randomly selected 1000, 2000, 5000, and 10,000 samples out of the original SNP data sets for APD analysis. We also created two new Hordeum vulgare and Glycine max data sets with the original samples, albeit having only 1000 randomly selected SNPs. The data sets with reduced SNP numbers served as the new whole data sets and were subjected to the same sampling scenarios as the original whole data sets, facilitating a comparative assessment of the effects of sample size and SNP number. For the number of SNPs, we generated new data sets with 2000 to 10,000 SNPs from Hordeum vulgare, Triticum aestivum-f20K, and Cicer arietinum-f300k data sets. For minor allelic frequency, we used Hordeum vulgare and Cicer arietinum data sets with minor allelic frequencies as low as 0.001 and generated new data sets by removing SNPs with minor allelic frequencies from 0.001 to 0.05 in these three original data sets. For missing SNP data, we selected Hordeum vulgare, Triticum aestivum-f20k, and Cicer arietinum-f300k data sets with different missing SNP data profiles and generated new data sets by removing SNPs with missing rates up to 0.07. For the APD analysis in each scenario, the APD.r script was modified as described in the Supplementary Materials C section. A new custom R script was specifically written to analyze and plot the APD correlations in each scenario.

## 3. Results

### 3.1. Variability of APD Estimates for 12 Germplasm Data Sets

The 12 germplasm SNP data sets (Table S2) represented different species of variable ploidy levels using different sequencing technologies by different bioinformatics tools. Supplementary Figure S1 showed the variability and heterogeneity of these data sets with respect to allelic frequency, minor allelic frequency, and missing SNP data. It is clear that the distributions of allelic frequencies, minor allelic frequencies, and missing data differed among the data sets. For example, there was a U shape, an L shape on the right, or an L shape on the left of the allelic frequency distributions (Figure S1). The distributions of minor allelic frequencies also varied, although a majority displayed the L shape on the left. These data sets also showed distributions of missing data with mainly the L shape on the left and could approach a missing data rate of 0.95 for some SNP loci.

**Figure 1:**
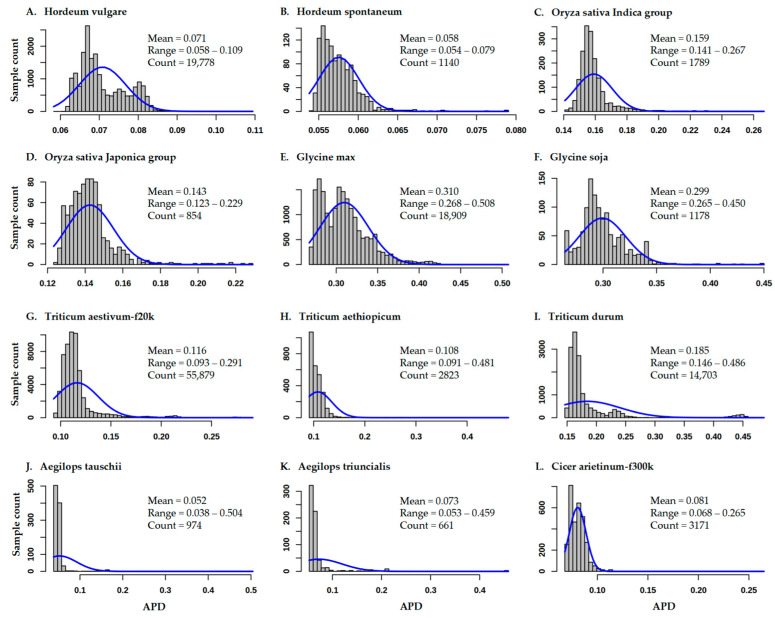
Distributions of APD estimates for all the samples in 12 germplasm APD data sets (**A**–**L**).

APD estimate for each sample in each data set was obtained, and these APD estimates were listed in 12 excel files (as listed in Supplementary Materials). The APD estimates displayed different frequency distributions across the 12 data sets (Figure 1). For example, most of the frequency distributions were skewed to the left, particularly for three *Triticum* spp. and two *Aegilops* spp. data sets, and there were APD estimates larger than three standard deviations (SD) on the right. Typically, the distribution for the Triticum durum data set revealed many estimates of less than two SDs on the left and larger than three SDs on the right, and the distribution for the Cicer arietinum-f300k data set displayed many estimates smaller or larger than two SDs on both the right and left. For specific data sets, a wide range of APD estimates was observed (Figure 1). For example, the APD estimates in Cicer arietinum-f300k and Triticum durum had a mean of 0.081 with a range of 0.068 to 0.265 and a mean of 0.185 with a range of 0.146 to 0.486, respectively. 

Given the wide distribution of the acquired APD estimates, we further characterized the ADP variations by grouping the assayed samples into six groups based on the distribution of ADP estimates: 1 (M + 3SD), 2 (M + 2SD), 3 (M + SD), 4 (M), 5 (M − SD), and 6 (M − 2SD). This APD-based grouping helped to group samples with different levels of genetic diversity in each data set (Table 1). Specifically, the grouping generated among-group SNP variances ranging from 1.52% (Oryza sativa Indica group) to 53.37% (Triticum triuncialis). Within each data set, the first APD-based group mostly displayed the least or negative group-specific Fst estimate (Table 1), suggesting the group had more rare alleles and thus more diversity than the other groups [36]. For example, the first groups in the two Oryza sativa data sets had the largest negative group-specific Fst estimates of −0.0499 and −0.0551. 

### 3.2. Genetic Outliers, Genetically Distinctive and Redundant Sets

The genetic outliers were identified and presented in each APD excel file (Supplementary Materials A1 section). Specifically, there were 291 samples with APD estimates larger than 0.2214 (or five standard deviations) in Triticum aestivum-f20k; 22 with APD > 0.2447 in Triticum aethiopicum; 17 with APD > 0.4625 in Triticum durum; 9 with APD > 0.2528 in Aegilops tauschii; 8 with APD > 0.3081 in Aegilops triuncialis; 15 with APD > 0.2171 in Oryza sativa Indica group; 7 with APD > 0.2050 in Oryza sativa Japonica group; 8 with APD > 0.4554 in Glycine max; 5 with APD > 0.4074 in Glycine soja; 7 with APD > 0.0700 in Hordeum spontaneum; 4 with APD > 0.1003 in Hordeum vulgare; and 1 with APD > 0.1221 in Cicer arietinum-f300k.

We also identified one genetically distinct group and one genetically redundant group of 100 samples each for each data set and listed them in each APD excel file. Examining the passport and related information in the published inventory, one could consider the redundant group as the genetically duplicated samples. For example, the redundant group of 100 cultivated soybean accessions was mainly acquired from South Korea and Japan, and 48 of these accessions have 99.9% similarity to at least one or other soybean accessions [25]. The redundant groups in Triticum aestivum and Hordeum vulgare data sets originated from Mexico and India with 77 breeding elite lines and from 17 countries with 41 breeding lines, respectively. In contrast (and interestingly), the redundant groups in the Triticum durum and Cicer arietinum data sets had 78 and 84 landrace samples, respectively. Similarly, based on the information available in the published inventories, the genetically distinct groups had diverse origins in the assayed samples. The distinct groups in the Hordeum vulgare and Glycine max data sets originated from 23 countries with 79 landraces and from 15 countries with 19 landraces, respectively. Note that some distinct groups may also include outliers (or the first APD-based group) for some data sets. Both distinct and redundant groups can be expanded, if needed, by selecting those samples with the highest or lowest APD estimates.

### 3.3. Variability of APD Estimation

**Figure 2:**
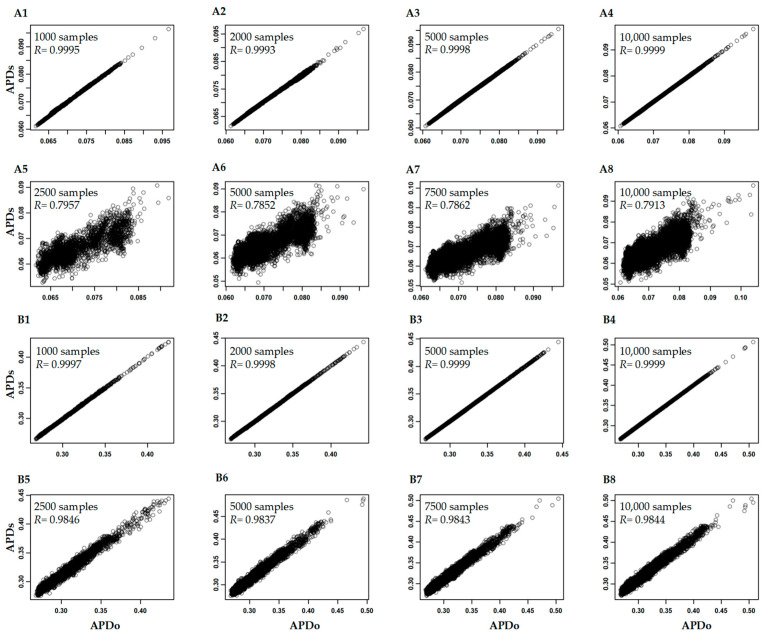
The correlations of APD estimates in the Hordeum vulgare (**A**) and Glycine max (**B**) data sets with all the original SNPs (**A1**–**A4**,**B1**–**B4**) or randomly selected 1000 SNPs (**A5**–**A8**,**B5**–**B8**) for all the original samples (APDo) and subject to the random selection of 1000 to 10,000 samples (APDs).

We analyzed the variation of APD estimation associated with four variables: sample size, SNP number, minor allelic frequency, and missing SNP data. By randomly sampling 1000, 2000, 5000, and 10,000 samples out of the original large Hordeum vulgare and Glycine max data sets, we found that the APD estimates had extremely high correlations of 0.999 or larger with the APD estimates in the original data sets (Figure 2(A1–A4,B1–B4)). However, if the original SNP data sets were reduced with only 1000 randomly selected SNPs, the reduced sample sizes were found to yield lower APD correlations. Specifically, for the Hordeum vulgare and Glycine max data sets, the correlations were roughly 0.79 (Figure 2(A5–A8)) and 0.98 (but with larger variances; Figure 2(B1–B4)), respectively. These results indicate that APD estimation was more sensitive to SNP number than the sample size.

**Figure 3:**
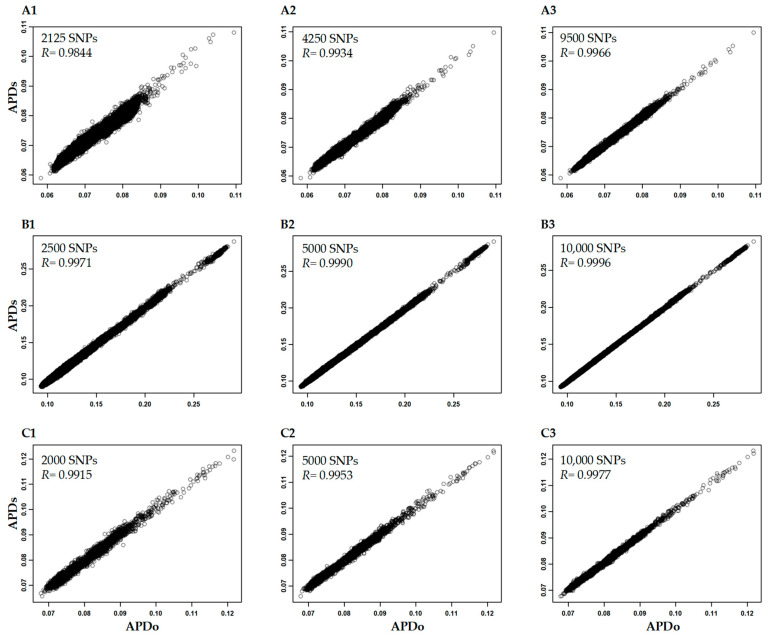
The correlations of APD estimates in the Hordeum vulgare (**A**), Triticum aestivum-f20k (**B**), and Cicer arietinum-f300k (**C**) data sets for all the original SNPs (APDo) and subject to the random selection of 2000 to 10,000 SNPs (APDs).

By randomly sampling 2000 to 10,000 SNPs from the Hordeum vulgare, Triticum aestivum-f20K, and Cicer arietinum-f300k data sets, we found that the revealed APD correlations associated with these smaller, variable SNP numbers were really high (>0.98) (Figure 3). However, the APD estimates in the Hordeum vulgare and Cicer arietinum data sets with SNP numbers less than 5000 still had large variance. These results indicate that 5000–10,000 genome-wide SNPs should be sufficient for an APD estimation.

**Figure 4:**
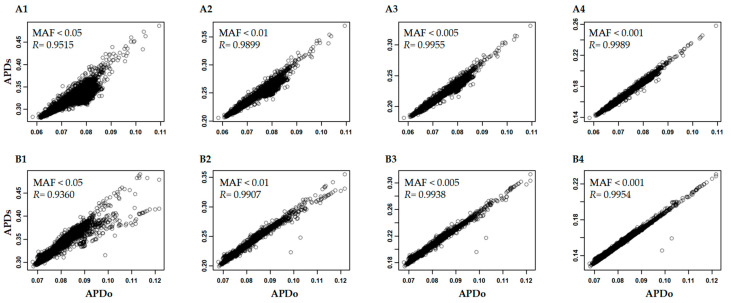
The correlations of APD estimates in the Hordeum vulgare (**A**) and Cicer arietinum-f300k (**B**) data sets for all the original SNPs (APDo) and subject to removal of SNPs with minor allelic frequencies (MAF) from 0.001 to 0.05 (APDs).

For variable SNP minor allelic frequencies. Figure 4 illustrates that the APD correlations remained high (or larger than 0.93) after the removal of SNPs with minor allelic frequencies from 0.001 to 0.05 in these three data sets and more variation in APD estimation was associated with the removal of SNPs with higher minor allelic frequencies. Particularly, the large variation in APD estimation was observed in either data set after removing SNPs with minor allelic frequencies smaller than 0.05 (Figure 4(A1,B1)), and consequently, the APD-based sample ranking may deviate more from the original sample ranking when removing SNPs with higher (up to 0.05) minor allelic frequencies. These results indicate that APD estimation was highly sensitive to the removal of SNPs with lower minor allelic frequencies.

**Figure 5:**
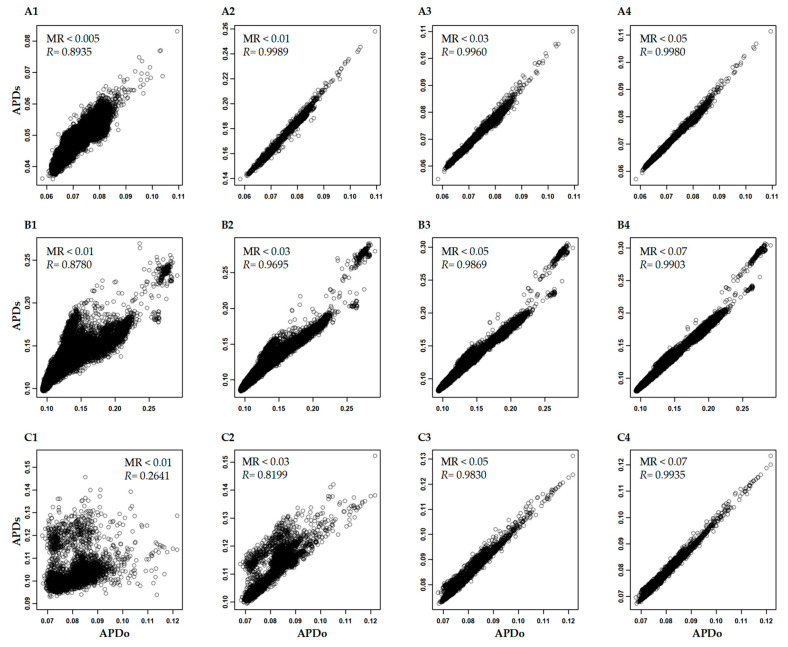
The correlations of APD estimates in the Hordeum vulgare (**A**), Triticum aestivum-f20k (**B**), and Cicer arietinum-f300k (**C**) data sets for all the original SNPs (APDo) and subject to removal of SNPs with missing data of rate (MR) 0.005 to 0.07 (APDs).

The impacts of missing SNP data on APD estimation (shown in Figure 5) varied greatly among the three data sets and were dependent not only on the extent of missing data but also on the pattern of missing data. For example, the APD estimation in the Hordeum vulgare data set had a correlation coefficient of 0.99 or larger when the SNPs with missing rates of 0.01 or larger were removed. In contrast, the APD estimations in both the Triticum aestivum-f20k and Cicer arietinum-f300k had a correlation coefficient of 0.99 or larger when the SNPs with missing rates of 0.07 or larger were excluded. Such differences among the data sets were expected, as the different missing rates were needed to remove most of the SNPs with missing data, as shown in Figure S1 (C3,E3,H3).

## 4. Discussion

This assessment revealed several significant findings for plant germplasm characterization and management, particularly in the genetic categorization of conserved germplasm. First, an APD assessment of large germplasm collections with published large genomic data sets was technically and practically feasible. Second, our assessment generated an APD estimate for each sample, a genetic outlier group, and genetically distinct and redundant groups of 100 samples each in the 12 germplasm APD data sets. The APD-based grouping revealed among-groups variances from 1.52 to 53.37% in various data sets. Third, the APD estimation was found to be more sensitive to SNP number, minor allele frequency, and missing data. An accurate sample APD estimation required 5000 to 10,000 genome-wide SNPs.

The assessment of APD variation yielded some encouraging findings for plant germplasm characterization. First, the custom APD.r script was capable of handling large genomic SNP data sets such as Triticum aestivum-f20k with 55,879 samples with 20,000 SNPs and Hordeum vulgare with 19,778 samples with 76,102 SNPs and had computation time up to a few days in a high-performance Linux server with up to 30 threads (Table S2). Such performance is highly significant, as an APD analysis of large data sets was shown to be technically and practically feasible. Second, our assessment also generated some useful data processing procedures to format different types of genomic SNP data (see Supplementary Materials B section), making the APD analysis more accessible. Third, it was found that SNP number, minor allele frequency, and missing data can affect the accuracy of the sample APD estimation. Thus, some consideration needs to be taken in an APD analysis. Removing SNPs with minor allelic frequencies <0.001 (Figure 4) and missing data rates from 0.05 to 0.07 (Figure 5) still showed high APD correlations with those in the original data sets and should not alter the APD-based sample ranking much. Generally, 5000 to 10,000 genome-wide SNPs are required for an accurate APD estimation of a sample against other samples (Figure 3). A sample with an accurate APD estimate will have an accurate APD-based ranking in either the whole or part of the assayed samples, as illustrated in Figure 2, although the scale of its sample ranking will differ between different sample sizes.

Our empirical assessment of APD variation was preliminary and further explorations still need to enhance the application of the APD approach to large and new genomic data. For example, we considered only the extent of the minor allelic frequency and missing SNP data, but not the variation patterns across the 12 data sets (Table S2). It is highly possible that different variation patterns may affect the APD estimation differently. Our APD.r script did not perform any imputation for missing data and considered only SNPs without missing data to calculate the pairwise dissimilarity. Further assessment is needed to compare the differences in APD estimation with and without imputation. The execution of the APD.r script for a large data set can last for days (Table S2). Thus, it is still desirable to improve pairwise sample computing with advanced algorithms to deal with an increasing number of samples and SNPs. We did not examine the impact of SNP ascertainment bias [37], nor linkage disequilibrium [38], on APD estimation. Theoretically, these factors can affect the APD estimation. For example, SNPs that are in proximity could bias an APD estimate by simply measuring the same haplotype blocks. As more pangenome data of plant germplasm will be generated in the coming decades, structural variants will become an important data type for the APD application to characterize genetic uniqueness, distinctness, and redundancy. Genetic variants of any nature associated with specific genes or phenotypic traits of interest may be more informative for the genetic categorization of plant germplasm and germplasm uses. How effective and informative a sample APD estimation is if based on new genetic and structural variants, however, remains to be studied.

The APD approach can assist in the assessment of genetic distinctness and/or redundancy in plant germplasm characterization. The assessment outputs (as listed in 12 APD excel files) can facilitate germplasm management at the five genebanks for uses such as the development of germplasm core subsets and accession identification for safety backup. However, the APD approach per se is of limited resolution in defining genetically redundant or distinct groups, as clearly stated in Fu [13]. There is no definite criterion that one could develop and apply with APD estimates to identify genetically redundant or distinct groups. This partly explained why the APD-based grouping was applied in this assessment to understand how large SNP variances could be accounted for by the first (or most distinct) and the last (or most redundant) groups. As Yang and Fu [19] suggested, additional information generated from iterative AMOVAs and PCoA plots could also be useful to identify a tentative group of genetically distinct accessions. Thus, caution is advised if the tentative group of genetically distinct accessions is directly used as a core subset, as it differs from the core collection defined by Frankel [39] and Brown [40] for germplasm management and utilization. On the other hand, the tentative redundant group can be used in combination with passport, evaluation, and characterization data to assist in the identification of truly duplicated accessions in a germplasm collection. In short, these tentative distinct and/or redundant groups can serve as a guide to facilitate plant germplasm management, utilization, and conservation. However, it is worth noting that the published genomic data (Table S1) sampled only an individual sample to represent a germplasm accession, but such a representation per se can be biased, as is the sample APD estimate reported here, particularly for heterogeneous accessions such as those of landraces even of self-fertilizing crops.

The revealed APD profiles in these 12 germplasm APD data sets helped to identify a genetic outlier group of samples with APD estimates larger than five standard deviations in each data set. Biologically, these outliers may suggest the presence of species’ misclassification, introgressed or hybrid germplasm. For example, the only Cicer arietinum sample identified as a breeding line from East Africa had an APD estimate of 0.2649, while the mean APD for the whole data set was 0.0806. This result strongly suggests that this sample may be misclassified as Cicer arietinum. There were 291 of 55,879 samples identified as outliers for the Triticum aestivum data set, and it is possible that these “outliers” may represent introgressed or hybrid germplasm. However, it is important for a genebank manager to assess the biological identification and status of these outliers, as our APD analysis can reveal only their extreme SNP variations but not verify their biological identity and status.

With APD estimates acquired at the individual sample level, it is feasible to perform the genetic categorization of conserved germplasm within a crop gene pool. An APD estimate represents another genetic diversity indicator associated with genetic distance and a sample with a higher APD estimate may harbor more rare and/or unique alleles than other samples with lower APD estimates. Thus, the APD estimate per sample can be used to genetically compare and group germplasm. The APD-based germplasm grouping following the APD distribution performed in this assessment provided an objective genetic tool to categorize conserved germplasm with up to six groups in each germplasm APD data set. As shown in Table 1, the APD-based grouping was genetically informative and captured considerable among-group SNP variances ranging from 1.52 to 53.37%. The first and second APD-based groups could also be served for the initial screening of germplasm with traits of interest if there is no trait-specific core subset in a germplasm collection [41]. With more genomic SNP data published on conserved germplasm, APD estimation will become more feasible and APD estimates are more accessible to be searched for genetically distinctive, redundant, or unique germplasm. It can be reasoned that APD-based categorization may be more informative at the individual sample level than those generated by other genetic clustering tools such as principal component analysis, as the latter mainly displays the overall variation pattern of all the assayed samples. To some extent, APD estimates may also be more informative to germplasm use than the sample heterozygosity estimates, as the former carries genetic information across a whole sample set. However, the extent of its informativeness still needs to be studied further, particularly in comparison with other genetic measurements. Additionally, APD-based categorization can be pursued together with phenotypic categorization via high-throughput phenotyping in a genebank to further enhance germplasm use.

## 5. Concluding Remarks

This assessment generated an APD estimate per sample, identified a genetic outlier group, and produced genetically distinct and redundant groups of 100 samples each in 12 germplasm APD data sets. The APD-based germplasm grouping revealed among-group variances ranging from 1.52 to 53.37% across these data sets. Further assessments revealed that these APD estimations were more sensitive to SNP number, minor allele frequency, and missing data. An APD assessment of large germplasm collections with published large genomic data sets was demonstrated to be technically and practically feasible. These findings together are useful for plant germplasm characterization and management, particularly in the genetic categorization of conserved germplasm.

## Figures and Tables

**Table 1:** APD grouping of the samples in 12 germplasm APD data sets and their diversity characteristics.

	Among-Group Variance	Group−Specific Fst and Group Size					
Data Set	(%) and Sample Size	1 (M + 3SD)	2 (M + 2SD)	3 (M + SD)	4 (M)	5 (M − SD)	6 (M − 2SD)
Oryza sativa Indica group	1.52	−0.0499	0.0224	−0.0606	−0.0215	0.0499	0.2610
	1789	38	29	87	430	1162	43
Oryza sativa Japonica group	13.17	−0.0551	−0.0487	0.1555	0.0768	0.1547	0.6476
	854	15	16	56	281	403	83
Glycine soja	11.92	−0.1049	0.0142	0.0479	0.0922	0.1677	0.7334
	1178	11	16	133	318	589	111
Glycine max	10.51	0.1773	0.0468	0.0723	0.1108	0.2325	0.4461
	18,909	295	480	2087	5373	7574	3100
Hordeum spontaneum	4.78	0.0619	0.0388	−0.0126	−0.0074	0.0528	0.2111
	1140	20	16	89	354	599	62
Hordeum vulgare	18.66	0.0677	0.1062	0.0850	0.0671	0.1938	0.5422
	19,778	32	328	3783	3336	9566	2733
Triticum aestivum-f20k	12.62 *	−0.2431	0.0243	0.0727	0.2105	0.3312	0.7615
	24,847 *	1657	682	2143	11,703	39,517	177
Triticum durum	25.39	0.0129	0.0621	0.3753	0.2783	0.4692	
	14,703	560	34	395	2265	11,449	
Triticum aethiopicum	22.07	−0.5228	0.1344	0.3092	0.4592	0.5490	
	2822	35	13	46	912	1816	
Aegilops tauschii	40.18	−0.4481	0.5697	0.4207	0.6904	0.7273	
	974	12	12	4	173	773	
Aegilops triuncialis	53.37	−0.1295	0.4138	0.4328	0.5576	0.6592	
	661	11	18	10	47	575	
Cicer arietinum-f300k	19.02	0.0579	−0.0310	0.0522	0.1536	0.3037	0.5342
	3171	31	63	273	1176	1279	349

* AMOVA for the among-group variance was made on only 24,847 Triticum aestivum samples that were randomly selected to represent the six APD-based groups due to the size limitation (40,000 samples) defaulted for the dist() function of R ad4 package.

## Data Availability

The data presented in this study are available in Supplementary Materials.

## Supplementary Materials

The complete Supplementary Materials (A–G below) can be accessed from Figshare DOI (https://doi.org/10.6084/m9.figshare.22143443). A. List of excel files for 12 germplasm APD data sets and five other supplementary files. B. Supplementary Materials and methods. C. Analysis of APD estimation with large genomic data. D. Associated files for computational analysis. E. Reference used in this supplementary file. F. Supplementary Tables: Table S1: The published genomic SNP data sets acquired from five major seed genebanks (CIMMYT, IPK, USDA-ARS, IRRI, and ICRISAT); Table S2: 12 specific cleaned data sets, approximate processing times, and overall average pairwise dissimilarity (APD). G. Supplementary Figure: Figure S1A: Frequency distribution of all the SNP alleles, minor alleles, and missing data for domesticated soybean (A), wild soybean (B), domesticated barley (C), and wild barley (D) SNP data sets; Figure S1B: Frequency distribution of all the SNP alleles, minor alleles, and missing data for hexaploid wheat (E), durum wheat (F), Triticum aethiopicum (G), and chickpea (H) SNP data sets; Figure S1C: Frequency distribution of all the SNP alleles, minor alleles, and missing data for Aegilops tauschii (I), Aegilops triuncialis (J), Japonica rice (K), and Indica rice (L) SNP data sets.
